# High-Visibility Photonic Crystal Fiber Interferometer as Multifunctional Sensor

**DOI:** 10.3390/s130202349

**Published:** 2013-02-08

**Authors:** G.A. Cárdenas-Sevilla, Fernando C. Fávero, Joel Villatoro

**Affiliations:** 1 Corning Cable Systems S.A. de C.V. Reynosa Plant, Cd. Reynosa, Tamaulipas 88730, Mexico; 2 Institute of Photonic Technology, Albert Einstein Str. 9, Jena 07745, Germany; E-Mail: fernando_favero@hotmail.com; 3 Aston Institute of Photonic Technologies, Aston University, Aston Triangle, Birmingham B4 7ET, UK; E-Mail: joel.villatoro@gmail.com

**Keywords:** interferometry, modal interference, photonic crystal fibers, refractive index sensors, liquid level sensors

## Abstract

A photonic crystal fiber (PCF) interferometer that exhibits record fringe contrast (∼40 dB) is demonstrated along with its sensing applications. The device operates in reflection mode and consists of a centimeter-long segment of properly selected PCF fusion spliced to single mode optical fibers. Two identical collapsed zones in the PCF combined with its modal properties allow high-visibility interference patterns. The interferometer is suitable for refractometric and liquid level sensing. The measuring refractive index range goes from 1.33 to 1.43 and the maximum resolution is ∼1.6 × 10^−5^.

## 1. Introduction

Due to their compactness, simplicity and high sensitivity, optical fiber modal interferometers have gained considerable attention by the sensor community. Modal interferometers exploit the relative phase displacement between two modes, typically two core modes or a core and a cladding mode. To fabricate a modal interferometer one needs a mechanism to couple light from the core into two or more modes and another mechanism to recombine them. To this end, different approaches have been proposed including for example, combination of different optical fibers [1–3], tapering techniques [4–6], or long period gratings [7,8]. Another approach consists of collapsing the voids of a photonic crystal fiber (PCF) over a microscopic region [9–11]. Such a process can be carried out by fusion splicing a PCF with a standard optical fiber [10,11]. The advantages of PCF modal interferometers built via voids collapsing are compactness, low temperature sensitivity, broad operation wavelength range, and high stability over time. All these properties are important for unambiguous measurement of the interferometer phase, and hence, the parameter being sensed.

The applications of optical fiber modal interferometers as sensors basically depend on the types of modes that participate in the interference. For example, cladding modes are sensitive to the external environment, thus diverse sensors can be implemented such as refractive index sensors and refractometers [2–8,10,11]. To enhance the sensitivity of such sensors it is desirable that the cladding mode is powerful, as this is the sensing mode. A good indication of the power of the interfering modes in a modal interferometer is the visibility or fringe contrast of the interference pattern. For instance, a relatively weak cladding mode gives rise to interferometers with low visibility. In modal interferometers based on standard fibers it is difficult to control the power of the interfering modes, hence the fringe contrast of the interference pattern.

In this paper we propose a PCF mode interferometer for index and level sensing. Our device is simple, robust, compact and reproducible. The interferometer operates in reflection as well as in transmission mode and consists of a centimeter-long stub of commercially-available PCF fusion spliced to standard optical fiber (SMF-28). Two identical collapsed zones in the PCF combined with the PCF geometry allow the efficient excitation and recombination of a core and a cladding mode in the PCF. This makes the device reflection spectrum exhibit well-defined interference patterns with a record fringe contrast (in excess of 40 dB). In our devices changes in the refractive index or liquid-level cause a detectable shift in the interference pattern. The index of refraction can also be measured by monitoring the power at a fixed wavelength. The refractive index resolution of our interferometers is between ∼1.6 × 10^−5^ and ∼7 × 10^−6^, thanks to the high visibility of the interference pattern. The maximum measuring range goes from 1.33 to 1.43. We believe that the device proposed here can be utilized in many refractometric-based sensing applications.

## 2. Device Design and Working Principle

**Figure 1.**
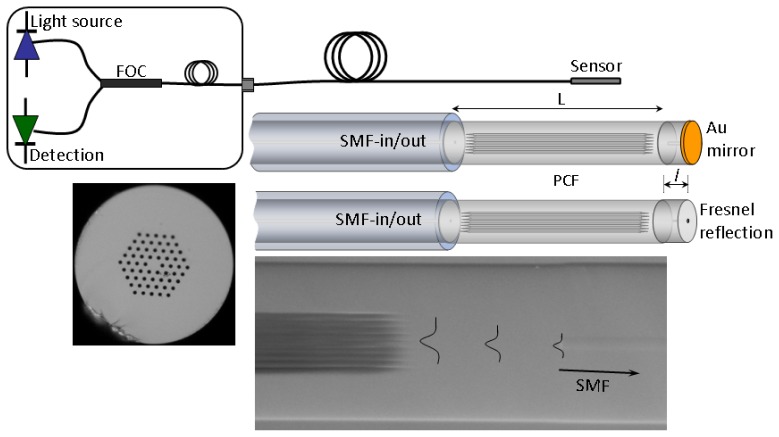
Drawings of our interferometer showing two possible terminations and schematic representation of the interrogation set up. FOC stands fiber optic circulator; SMF for single mode fiber, L for PCF length, and *l* is the length of SMF at the distal end. The micrographs show the PCF cross section and details of the PCF-SMF junction. The broadening of the beam when it enters the collapsed region is illustrated.

The cross section of the PCF used to fabricate the interferometers is shown in Figure 1. The PCF is commercially available, has six-fold symmetry in the void structure and is known as large-mode-area (LMA-10) PCF. The parameters of such a fiber are the following: core size diameter, 10 μm, average diameter of the voids, 3.1 μm, and average separation between the voids (pitch), 6.6 μm. The modal properties of this type of PCF have been reported elsewhere [12]. The fabrication of the devices here proposed only involves cleaving and fusion splicing; this ensures high reproducibility and low cost. Any commercial cleaving and splicing machines can be used. If the SMF and the LMA-10 PCF are spliced with a default program set in the machine for splicing single- or multi-mode fibers, the PCF's air holes will entirely collapse over a length of a few hundred micrometers [11]. However, by adjusting the intensity and duration of the arc discharge of the fusion process the length of the collapsed zones can be controlled [13]. To achieve interferometers with high visibility both splices need to be identical (unequal splices give rise to devices with completely different properties, see Reference [14]). It was found experimentally that the length of the collapsed zones could be from ∼150 μm to ∼220 μm, other lengths gave rise to interferometers with lower visibilities.

The proposed interferometer is sketched in Figure 1 along with a schematic diagram of the interrogation system. The interferometer was intended to operate in reflection mode. To avoid issues imposed by the reflection of cladding modes in a PCF, see for example [10], a short section of SMF was spliced to the PCF at the distal end. In this way the PCF voids at the end were completely sealed. In this regard, our devices are different to those reported in [15]. The length of the SMF at the distal end can range from ∼2 mm to tens of centimeters and even several meters. The SMF at the tip must be cleaved and coated with a highly reflecting mirror. Although a cleaved fiber end without any coating can also be used as a mirror (due to Fresnel reflection).

Note from Figure 1 that the outer diameters of the SMF and the PCF in our interferometer are permanently aligned. This means that there is axial symmetry in the structure. On the other hand, the collapsed zones in the PCF cause a broadening of the beam when it propagates from the SMF to the PCF [10,11]. The broadening of the beam combined with the axial symmetry and the modal properties of the PCF are what allow the excitation (and recombination) of modes that have similar azimuthal symmetry [14]. The modes excited in the PCF have different effective indices (or different propagation constants), thus they travel at different speeds. As a result, the modes accumulate a phase difference as they propagate along the PCF. Due to the excitation and recombination of modes in the device, the reflection spectrum is expected to exhibit a series of maxima and minima (interference pattern). When two modes participate in the interference the transmitted or reflected intensity (*I*) can be expressed as:

(1) $$I=I_{1}+I_{2}+2\sqrt{I_{1}I_{2}}\cos(\Delta \phi ).$$

In Equation (1)*I*_1_ and *I*_2_ are, respectively, the intensity of the core mode and the cladding mode and *ΔΦ* = *2πΔnL/λ* is the total phase shift. *Δn* = *n_f_* − *n_c_*, *n_f_* and *n_c_* being, respectively, the effective refractive index of the core mode and the cladding mode. L is the physical length of the PCF and *λ* the wavelength of the optical source. The fringe spacing or period (*P*) of the interference pattern is given by *P* = *λ*^2^/(*Δn*L). The maxima of the interference pattern appear at wavelengths that satisfy the condition *ΔΦ* = *2mπ,* with *m* = 1, 2, 3… This means at wavelengths given by

(2) $$\lambda_{\text{m}}=\Delta n\text{L}/m$$

**Figure 2.**
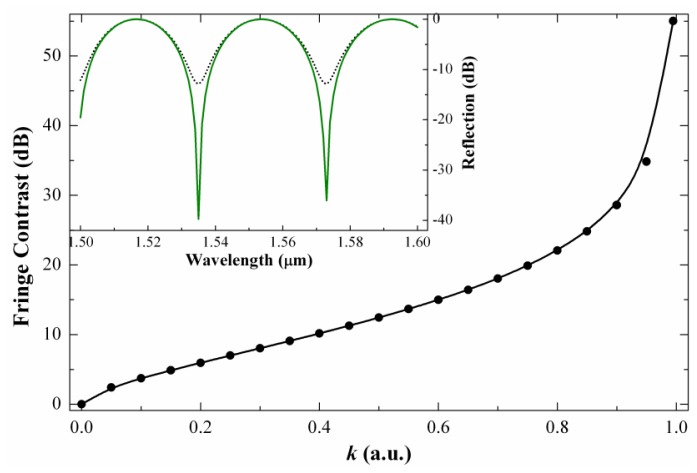
Fringe contrast in a mode interferometer as a function of *k* or the intensity of the cladding mode to that of the core mode ratio. The inset shows the theoretical reflection spectrum in the case of *k* = 0.4 (dotted line) and *k* = 0.96 (solid line).

The fringe contrast or visibility (*V*) of a modal interferometer is an important parameter, particularly when the interferometer is used for sensing applications. Typically, higher visibility is desirable since it leads to larger signal-to-noise ratio and more accurate measurement. The visibility of a two-mode interferometer can be calculated by the well-known expression: *V* = (*I*_max_ − *I*_min_)/(*I*_max_ + *I*_min_), where *I_max_* and *I_min_* are, respectively, the maximum and minimum values of *I* given in Equation (1). According to the definition and Equation (1)*V* can be expressed as [16]:

(3) $$V=2\sqrt{k}/(1+k).$$

where *k* = *I*_1_/*I*_2_. Many research groups prefer fringe contrast (expressed in dB) instead of visibility. The fringe contrast (*FC*) is defined here as *FC* = −10log(1−*V*). In Figure 2 we show the dependence of the fringe contrast on *k* along with the theoretical interference pattern of device with L = 10 mm for two values of *k*. It can be noted that the fringe contrast increases as *k* approaches to 1, *i.e.*, when the two modes that participate in the interference have equal intensities.

In our interferometer the length of the PCF can be controlled very precisely while the wavelength of the optical source can be chosen. The types of modes that participate in the interference and their intensities, *i.e.*, Δ*n* and *I*_1_ and *I*_2_ can eventually be controlled with the PCF geometry. Thus, it seems possible to fabricate mode interferometers with specific periods and visibilities.

**Figure 3.**
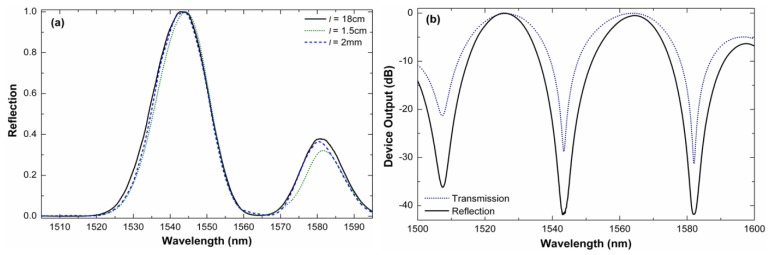
(**a**) Normalized reflection of a 12 mm-long interferometer for different lengths of SMF at the distal end. (**b**) Normalized transmission and reflection spectra of a 16 mm-long interferometer when a highly reflecting mirror was used. In all cases the external medium was air and the PCF was LMA-10.

It is important to point out that fusion splicing machines can be programmed to automatically align an SMF and an LMA-10 and apply the exact amount of heat to join permanently the two fibers together, thus ensuring high reproducibility. A collection of devices were fabricated with a commercial fusion splicing machine (Ericsson FSU 955) under the same splicing conditions. To interrogate the interferometers, light from an LED (peak power of 10 mW) was launched to them and the reflected light was fed to an optical spectrum analyzer by means of a fiber optic circulator. Figure 3(a) shows the normalized reflected power of a 12 mm-long interferometer observed for different lengths of SMF at the distal end. In these cases the light was bounced off the cleaved end by Fresnel reflection. The section of SMF at the distal end was coated with index matching gel to absorb or scatter possible cladding modes in the SMF. It can be noted that the length of SMF at the end does not affect the performance of the interferometer. It is important to mention that the reflected power at the minima was on the order of picowatts level due to the low reflectivity (less than 4%) of the cleaved fiber end, the insertion losses of the interferometer (around 6 dB). In a practical application the unprotected end can be an issue, but it can be overcome with a highly reflecting mirror. Figure 3(b) shows the spectra of a 16 mm-long interferometer when a commercially available fiber optic reflector (OZ Optics) was spliced at the distal end. The fringe contrast in this case exceeds 40 dB (V = 0.9999) which is considerably higher than that of any other mode interferometer reported until now. The modal properties of the PCF employed and the configuration of the device largely contributes to achieve well-defined interference patterns and high fringe contrast. For comparison, the transmission spectrum of the device was measured; it is shown in Figure 3(b). It can be noted that the period of the interferometer in both reflection and transmission modes is the same, however, the visibility is considerably higher when the device operates in reflection mode. We believe that these properties are due to the fact that the PCF cladding mode does not experience double pass along the PCF since it is not reflected from the mirror. The core mode does experience double pass in the PCF. Due to the two identical collapsed regions in the PCF and the short section of SMF at the distal end our device behaves in a completely different manner than that reported in Reference [15].

## 3. Results and Discussion

The excited cladding mode reaches the PCF-external-medium interface and becomes highly sensitive to the external refractive index (RI). Thus the interferometer can be exploited for refractometric sensing. The presence of a liquid or a layer on the PCF will change solely the effective index of the cladding mode since the core mode is completely isolated and it does not interact with the external medium. According to Equation (2) changes in *n_c_* hence in Δ*n*, will make *λ_m_* to shift by *Δλ_m_* provided that L is kept fixed. The position of the maximum or maxima of the interference pattern can be determined very accurately even with a low-cost spectrometer. To avoid a *Δλ_m_* = *mP* with *m* an enter, *i.e.*, shifts that are a multiple of the period of the interference pattern, compact devices were be fabricated.

**Figure 4.**
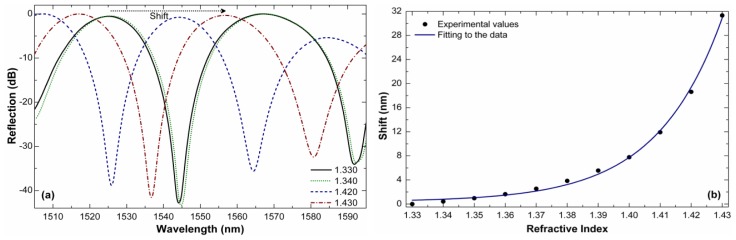
(**a**) Interference pattern of a 12 mm-long interferometer immersed in different indices. (**b**) Corresponding interference pattern shift *versus* RI. The measurements were carried out at room temperature with fluctuations on the order of 2 °C.

Figure 4(a) shows the reflection spectra (using the commercial reflector mentioned above) of a 12 mm-long device for indices in the 1.330–1.430 range. It can be noted that the shift in the whole measuring range is less that the interferometer period for which there is no ambiguity in the measurements. Note also that whatever the external index the interference pattern remains well defined and that the visibility does not change. Figure 4(b) shows the observed shift in our 12 mm-long interferometer as a function of the external index. It should be pointed out that a shift of 0 nm was assumed when the device was in water (RI of 1.333).

Like most fiber-based RI sensors, the maximum sensitivity of our interferometer is for higher indices. For example, the shift of the interference pattern when the external index changes from 1.420 to 1.430 is ∼12.70 nm, see Figure 4(b). This means that the sensitivity in that RI range reaches a value of ∼1270 nm/RIU (RIU refers to refractive index units). The resolution of our interferometer, *i.e.*, the smallest RI change it can detect, will depend on the minimum shift *Δλ*_m_ that can be detected. With simple peak-tracking algorithms [17] or by performing a Fourier transformation [18] *Δλ* on the order of ∼20 pm can be resolved; this means that a resolution of our RI sensor can reach ∼1.6 × 10^−5^. Such a resolution is an order of magnitude higher than that of interferometers built with LMA-8 PCF [11]. In addition, the present interferometer is between 3 to 10 times shorter than that reported in [11]. The results here presented suggest the importance of the PCF design and the configuration in which the interferometer operates.

**Figure 5.**
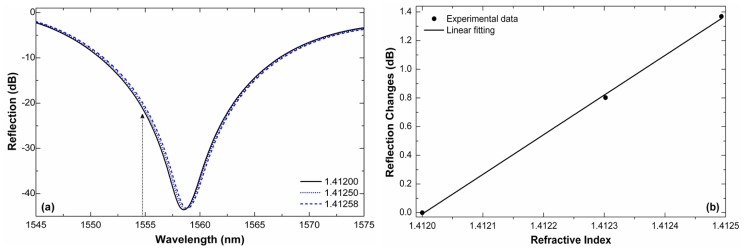
(**a**) Reflection spectra for three RI values that are closed to each other. (**b**) Reflection changes observed at λ = 1,554.8 nm as a function of the external RI. The interferometer was 12 mm long.

Figure 5(a) shows a close up of the minima of the interference patterns of a 12 mm-long interferometer for three RI values that are close to each other. The shift is minimal but quantifiable. If we monitor the power of the wavelength located at the point of maximum slope, or e.g., at *λ* = 1,554.8 nm (see the arrow in the figure) and by taking 1.4120 as reference, we can see that an increment of 5.8 × 10^−4^ in the refractive index causes a transmission change of nearly 1.4 dB. Large changes in the reflection are due to the high fringe contrast of the interferometer. Thus, a resolution of ∼7 × 10^−6^ can be achieved if transmission changes of 0.01 dB can be resolved. The resolution can be even higher for indices in the 1.420–1.430 range.

**Figure 6.**
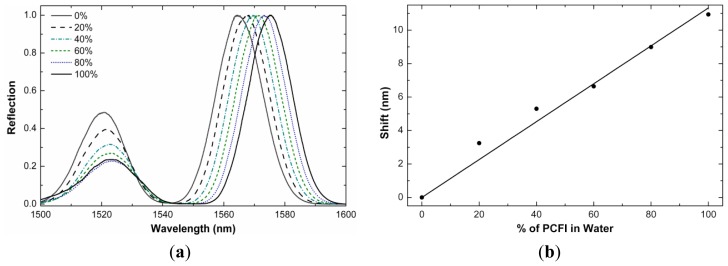
Reflection spectra of an interferometer at different percentages of the length of the PCF immersed in water and the corresponding calibration curve. The PCF length, L, was 11 mm.

The refractive index sensitivity of our interferometers can be exploited to form a liquid level sensor. We observed that the shift of the interference pattern of our interferometers was dependent on the fraction of the length of the PCF that was immersed in water (or any other liquid). In Figure 6(a) we show the interference patterns exhibited by a device (L = 11 mm) operating in reflection mode and immersed partially or totally in water. The remaining section of the PCF was in air. Note that the shift of the interference pattern increases with the percentage of the length of the PCF that is immersed in the liquid. Figure 6(b) shows the calibration curve. Our liquid-level sensor showed a large linear range with sensitivity of 0.11 nm shift per millimeter of PCF immersed in water. The sensitivity may be higher for liquids with high index as the shift is more prominent for liquids with high index of refraction, see Figure 4(b). As liquid-level sensors our interferometers can be an alternative to those based on gratings [19,20] or other approaches that are a bit more complex [21].

It is important to mention here that the above results were obtained at a fixed temperature with variations on the order of 2 °C. Higher temperature fluctuations may affect the performance of our interferometers as temperature modifies the propagation constant of the interfering modes and causes the interference pattern to shift. Our interferometer has a temperature sensitivity of ∼9 pm/°C and thus temperature fluctuations of around ∼6 °C can be tolerated to achieve the refractive index resolutions mentioned above. Another factor that can affect the resolution is the stability of the optical source during the measurements. Highly stable, single frequency and tunable lasers for the telecom wavelength range are commercially available. In addition, power fluctuations can be compensated easily. RI measurements in a fixed or controlled temperature environment are also feasible. Therefore, the aforementioned resolutions are reachable and the real-world applications of the interferometers here presented are promising.

## 4. Conclusions and Outlook

A compact, robust and simple photonic crystal fiber interferometer that operates in reflection mode was proposed for refractive index and liquid-level sensing. The device consists of a short section (in the 10−12 mm range) of commercially available PCF fusion spliced to single mode fibers. During the splicing process the voids of the PCF are intentionally collapsed over a microscopic region. The collapsed zone introduces an axial offset, and consequently, a mode field mismatch which allows the excitation and recombination of core and cladding modes in the PCF. Since the modes propagate at different phase velocity they accumulate a phase difference. As a result, the reflection spectrum of the device exhibits a sinusoidal pattern. Liquids or coating on the PCF surface modify the phase difference of the interfering modes, thus causing a shift of the interference pattern. The interferometer proposed here is more compact than others based on PCF or standard optical fiber. In addition, record fringe contrasts (∼40 dB) were observed.

The potential of our interferometer for refractometric sensing was demonstrated. The sensitivity to the external index of refraction was also exploited to form simple liquid-level sensors. Our results suggest that bulk RI changes on the order of 10^−5^ and 10^−6^ can be resolved, depending on whether one monitors the shift of the interference pattern or the reflected power at a fixed wavelength. Optimization of the PCF structure (holes diameter and separation between holes) may enhance the resolution even further. The high resolution of our interferometer can be particularly useful if one wants to monitor minute RI changes experienced, e.g., by a sensitive thin film or layer deposited on the PCF. The layer RI and its thickness can be tailored, thus, a number of chemical and biological sensors can be developed with the interferometer here proposed.
